# Production of fungal hypocrellin photosensitizers: Exploiting bambusicolous fungi and elicitation strategies in mycelium cultures

**DOI:** 10.1080/21501203.2024.2430726

**Published:** 2024-12-13

**Authors:** Xin Ping Li, Wen Hao Shen, Jian Wen Wang, Li Ping Zheng

**Affiliations:** a College of Pharmaceutical Sciences, Soochow University, Suzhou, China; b Department of Horticultural Sciences, Gold Mantis School of Architecture, Soochow University, Suzhou, China

**Keywords:** *Shiraia*, hypocrellins, endophytes, mycelium culture, elicitation

## Abstract

Hypocrellins, a group of naturally occurring perylenequinone pigments produced by *Shiraia bambusicola*, are notable for their potential use in photodynamic therapy (PDT) for treating cancers and viruses. Traditionally, hypocrellins have been extracted from the fruiting bodies of *S. bambusicola*, a parasitic fungus on bamboo. However, the yield from wild *Shiraia* fruiting bodies is often insufficient, prompting a shift towards seeking other fungi with higher yields of hypocrellins as alternative sources. This review comprehensively examines the current research on the isolation, identification, and bioactivity of fungal perylenequinones from *Shiraia* isolates from ascostromata or fruiting bodies, *Shiraia*-like endophytes, and other endophytes from bamboos. Additionally, the review discusses the culture methods and conditions for solid-state and submerged fermentation of hypocrellin-producing fungi, including medium components, culture conditions, and optimisation of fermentation factors, as mycelium cultures have emerged as a promising alternative for the production of hypocrellins. Furthermore, novel elicitation strategies are presented to address the bottleneck of lower production of hypocrellins in mycelium cultures, focusing on the preparation, characterisation, and application of biotic and abiotic elicitors. This review aims to facilitate further exploration and utilisation of fungal resources and elicitation strategies for enhanced production of hypocrellins in mycelium cultures.

## GRAPHICAL ABSTRACT

**Figure:**
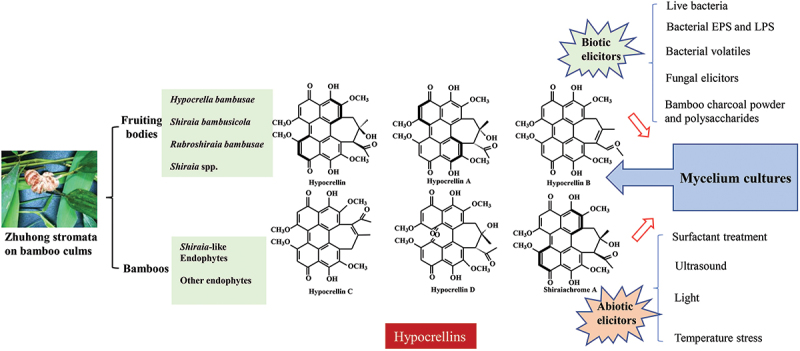

## 1. Introduction

**Figure 1.**
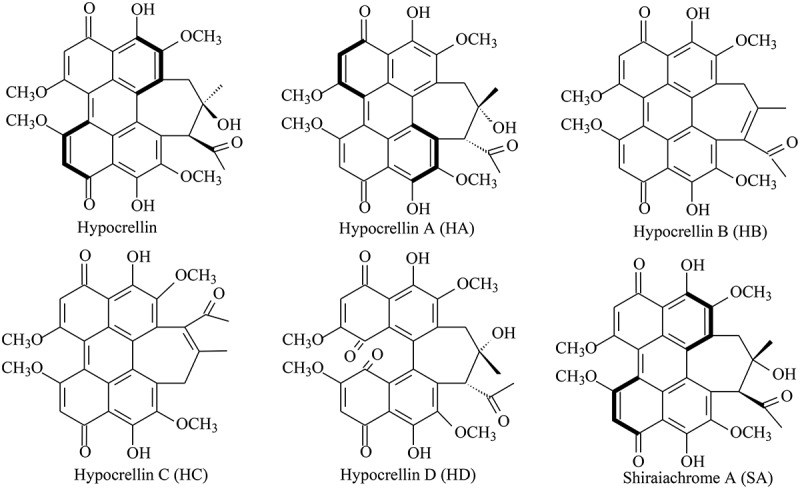
The chemical structure of hypocrellin and its derivatives.

**Figure 2.**
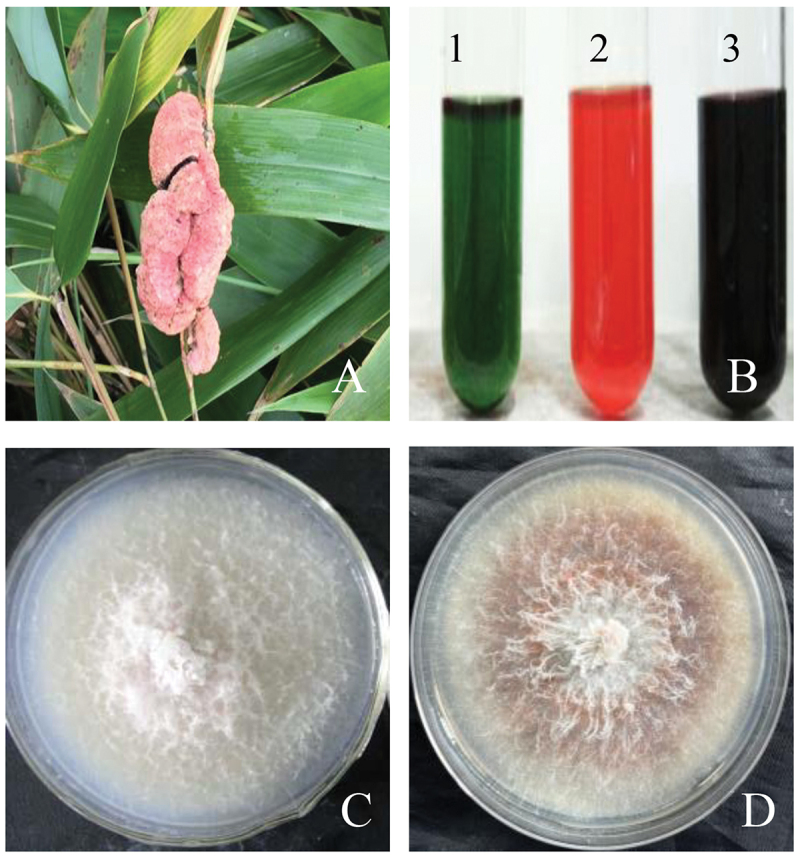
*Shiraia bambusicola* and the color reaction of hypocrellin pigments. (A) The pinkish *Shiraia* ascostromata on living bamboo branches. (B) The pigment acetone extract with addition of sodium hydroxide solution (1), hydrochloric acid solution (2), and FeCl_3_ solution at 1 mol/L (3), respectively. (C, D) Top view of non-hypocrellin producing strain (C) and hypocrellin-producing strain of *S. bambusicola* (D).

Hypocrellins are naturally occurring fungal perylenequinone pigments with potential photodynamic activities against cancer and microbial diseases, including hypocrellin, hypocrellin A–D (HA–HD) and shiraiachrome A–C (Figure 1). The hypocrellin family is distinguished by a helical chiral pentacyclic core fused with a C7, C7’-seven-membered carbocyclic ring and features centrochiral stereogenic centres (O’Brien et al. 2010). These perylenequinones share a fundamental 3,10-dihydroxy-4,9-perylenequinone-chromophore responsible for light absorption and subsequent generation of reactive oxygen species (ROS) such as hydroxyl radicals (•OH), superoxide anions (O_2_•^−^), hydrogen peroxide (H_2_O_2_), and singlet oxygen (^1^O_2_). Hypocrellins have garnered significant attention as promising photosensitisers within the perylenequinone group for photodynamic therapy (PDT), particularly in the treatment of skin diseases and cancers (Diwu 1995). These compounds are typically extracted from the hyphae, ascostromata, or fruiting bodies of *Shiraia bambusicola* P. Hennigs and related fungi (Wu et al. 1989; Kishi et al. 1991; Tong et al. 2021). *S. bambusicola*, a parasitic fungus found on living culms of *Brachystachyum* or *Pleioblastus* bamboos in temperate regions of China and Japan (Amano 1983; Liu et al. 2012b), produces large pinkish ascostromata on living bamboo branches (Figure 2). These ascostromatas, known as “Zhuhuang” in traditional Chinese medicine for centuries, are utilised in treating rheumatoid arthritis, sciatica, trachitis, febrile convulsion, and oxyhepatitis (Diwu 1995; Jia et al. 2006). Clinical applications of hypocrellins in China include the use of hypocrellin ointment to treat lichen amyloidosis, tinea capitis, white lesions of the vulva, vitiligo, psoriasis, and keloids (Wan and Chen 1981; Liang et al. 1984; Cui 2017; Wang et al. 2020; Guan 2021). Due to their high singlet oxygen quantum yield, low dark toxicity, strong red-absorption properties, and rapid tissue clearance, hypocrellins have garnered significant interest as potent photosensitisers for PDT in cancer (Diwu et al. 1994; Park et al. 1998; Ali et al. 2002; Kitamura et al. 2022; Liu et al. 2023; Yu et al. 2024) and viral infections (Hudson et al. 1994; Hirayama et al. 1997; Alferova et al. 2022). Additionally, hypocrellins exhibit antimicrobial and antileishmanial photodynamic inactivation properties (Ma et al. 2004; Bao et al. 2023), and potent immunomodulatory effects (Korbelik et al. 2009; Chen et al. 2011; Park et al. 2011). Their distinct fluorescent properties have positioned hypocrellins as viable fluorescent probe molecules in biomedical research (Diwu et al. 1989; Xu et al. 2004; Zhang et al. 2021). Moreover, due to their bright colours, strong antimicrobial activity, good dye affinity, and higher lipid solubility, hypocrellins hold promise as edible natural colourants or preservatives in food (Su et al. 2009, 2011; Shi et al. 2016).

In the past decade, significant advancements have been achieved in elucidating the bioactivity and biotechnological production of hypocrellin photosensitisers. Recent reviews by Khiralla et al. (2022) and Deng et al. (2022) have comprehensively outlined the occurrence, classification, biosynthesis, and bioactivities of fungal perylenequinones (66 compounds). Daub et al. (2005) provided insights into the biosynthesis and physiological roles of cercosporin, a well-studied perylenequinone toxin produced by *Cercospora* species. Chemical and physical properties of HA and HB were summarised by Diwu and Lown (1990). Moreover, Bao et al. (2023) reviewed recent publications on the biosynthesis and biotechnological production of hypocrellins. While hypocrellins have primarily been isolated from the fruiting bodies of *S. bambusicola*, the cultured strains often yield lower or negligible amounts of hypocrellins. Consequently, attention has shifted towards *Shiraia*-like endophytes, which exhibit promising capabilities for the production of hypocrellins (Morakotkarn et al. 2008; Liang et al. 2009b; Shen et al. 2014; Zhang et al. 2014; Tong et al. 2017). Despite various attempts to address the challenge of low productivity through elicitation approaches in previous studies, a dedicated review focusing on fungal resources for production of hypocrellins and elicitation strategies in detail is lacking. Hence, the objective of this paper is to provide a comprehensive review of recent research on hypocrellins-producing strains, the origin of hypocrellins, mycelium culture techniques, and elicitation strategies. This review will facilitate further exploration of fungal resources for hypocrellin production and enhance the utilisation of hypocrellins in photodynamic therapy in the future.

## 2. Fungal resources for production of hypocrellins

**Figure 3.**
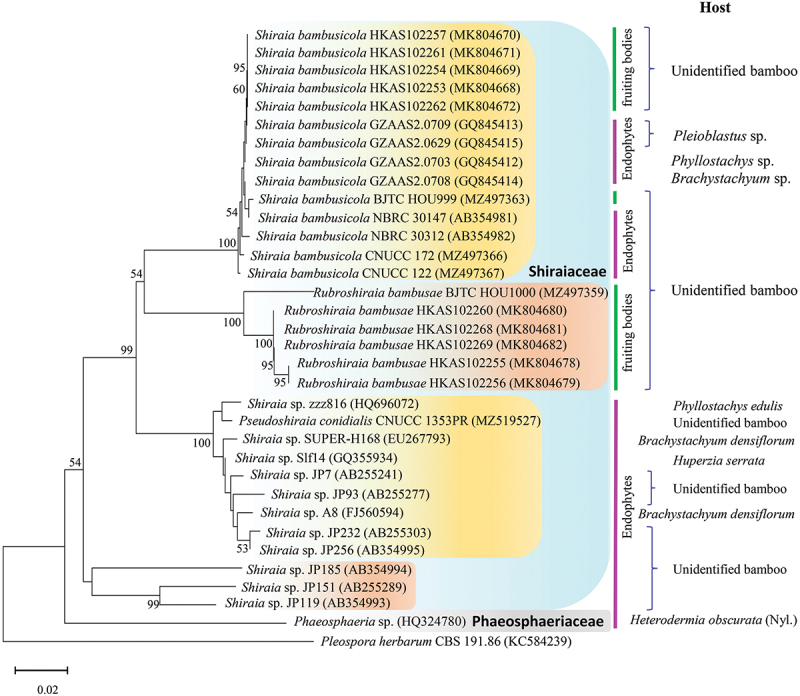
Maximum likelihood phylogenetic tree for hypocrellin-producing fungi generated from MEGA11, based on ITS sequences data. Confidence values above 50% obtained from a 1,000-replicate bootstrap analysis are indicated at the branch nodes. The scale bar indicates the number of estimated substitutions per site. *Pleospora herbarum* (CBS 191.86) was used as outgroup for rooting the tree. GenBank accession numbers are given parentheses.

The monotypic genus *Shiraia*, originally designated by the Japanese plant pathologist Mitsutaro Shirai, was initially proposed as a member of the family Nectriaceae in 1900, with *S. bambusicola* P. Henn identified as its type species (Hennings 1900). Subsequently, Saccardo (1902) relocated the genus *Shiraia* to the family Hypocreaceae (Hypocreales) due to the distinctive features of its large and persistent ascostromata. Over the past few decades, the genus was classified into Hypocreales, Pleosporales, Dothideales incertae sedis on the basis of its morphological characteristics (Teng 1934; Amano 1980; Zhang et al. 2012). However, molecular analyses utilising 18S rDNA and ITS-5.8S rDNA sequences conducted by Cheng et al. (2004) led to the classification of the genus *Shiraia* within the order Pleosporales. Subsequently, Liu et al. (2013) introduced the family Shiraiaceae within the order Pleosporales to accommodate the genus *Shiraia*, based on morphological characteristics and phylogenetic analyses of nuclear ribosomal DNA (nrDNA) sequences. Recently, Dai et al. (2019) described a novel genus, *Rubroshiraia*, within the family Shiraiaceae based on the morphological characteristics and phylogenetic analysis. Figure 3 shows the phylogenetic tree for hypocrellin-producing fungi.

### 2.1. Ascostromata or fruiting bodies

Hypocrellin is a dark red pigment with photodynamic activity isolated from *H. bambusae* ascostromata (Chen et al. 1981). HA, an enantiomer of hypocrellin, was also isolated initially from the ascostromata extracts (Wan and Chen 1981), which was renowned for its photodynamic activity against various Gram-positive bacteria (Table 1). HA has been isolated from fruiting bodies of *S. bambusicola* and referred by Wu et al. (1989) as shiraiachrome B. HA as the enantiomer of hypocrellin is the major perylenequinone constituent in the stromata extracts of *H. bambusae* (Li et al. 2021). Subsequently, HB was isolated from ethanol extracts, with its structure elucidated by Wan et al. (1985). HC was independently isolated from stromatal tissues of both *S. bambusicola* by Kishi et al. (1991) and *H. bambusae* by Zheng et al. (2010). The series of hypocrellin derivatives, denoted as HA-HD, were isolated from the fruiting bodies of *S. bambusicola* by Fang et al. (2006) and hypocrellins referred to the sum of such hypocrellin derivatives. Bioassay-guided fractionation of methanolic and acetone extracts of *S. bambusicola* mycelia led to the isolation of cytotoxic compounds known as shiraiachromes A, B, and C (Wu et al. 1989; Wang et al. 1992). High-performance liquid chromatography (HPLC) analysis revealed the simultaneous presence of hypocrellins A, B, or C, with contents ranging from 3.43–8.37 mg/g, 0.377–0.815 mg/g, and 0.487 to 0.950 mg/g, respectively, in the ascostromata of *H. bambusae* collected from various Chinese provinces (Kong et al. 2012). The fruit bodies of *S. bambusicola* BJTC HOU999, collected from Hangzhou, China, contained 3.6 mg/g HA, 1.80 mg/g HB, and 4.99 mg/g shiraiachrome A (SA), while those of *Rubroshiraia bambusae* BJTC HOU1000 from Yunnan, China, produced 49.54 mg/g HA, 6.02 mg/g HB, and 10.34 mg/g SA, respectively (Tong et al. 2021). However, hypocrellin compounds were not detected in extracts from the mycelia of both *S. bambusicola* and *R. bambusae*. Hu et al. (2008) isolated the *S. bambusicola* strain ZH-5-1 from *Shiraia* fruit bodies in Anhui, China, with the production of hypocrellins ranging from 0.05 mg/g to 2.94 mg/g in mycelium cultures.

**Table 1.** Fungal strains for the production of hypocrellins.

Fungal species		ITS numbers	Host plant	PQ contents	References
Ascostromata or fruiting bodies					
*Hypocrella bambusae*		–	–	HA	Wan and Chen (1981)
*H. bambusae*		–	–	HB	Wan et al. (1985)
*Shiraia bambusicola*		–	Bamboo	HA-30.8 mg/90 g air-dried stomata, HB-31.1 mg/90 g air-dried stomata, HC-30.4 mg/90 g air-dried stomata	Kishi et al. (1991)
*H. bambusae*		–	Bamboo	EA-15.3 mg/4.4 kg dry fruiting bodies, HA-772 mg/4.4 kg dry fruiting bodies, HB-96 mg/4.4 kg dry fruiting bodies, HC-173 mg/4.4 kg dry fruiting bodies, 1, 8-dihydroxy anthraquinone-7 mg/4.4 kg dry fruiting bodies	Zheng et al. (2010)
*S. bambusicola*		–	Bamboo	HA-42.3 mg/1.5 kg fruiting bodies, HB-21.5 mg/1.5 kg fruiting bodies, HC-19.6 mg/1.5 kg fruiting bodies, HD-15.5 mg/1.5 kg fruiting bodies	Fang et al. (2006)
*S. bambusicola*		–	Bamboo	Shiraiachromes A-27.2 mg/100 mg crude extract, Shiraiachromes B-24.4 mg/100 mg crude extract, Shiraiachromes C-5.1 mg/100 mg crude extract	Wu et al. (1989)
*S. bambusicola*		–	Bamboo	Shiraiachromes A and B	Wang et al. (1992)
*H. bambusae*		–	–	HA-3.43–8.37 mg/g, HB-0.377–0.815 mg/g, HC-0.487–0.950 mg/g	Kong et al. (2012)
*S. bambusicola* BJTC HOU999		MZ497363	–	HA-3.60 mg/g, HB-1.80 mg/g, SA-4.99 mg/g	Tong et al. (2021)
*Rubroshiraia bambusae* BJTC HOU1000		MZ497359	–	HA-49.54 mg/g, HB-6.02 mg/g, SA-10.34 mg/g	Tong et al. (2021)
Hypocrellin-yielding endophytes					
*Shiraia*-like endophytes	Strain g05 (JP7), g74 (JP232), g43 (JP93), and g58 (JP151)	AB255241 (JP7), AB255303 (JP232), AB255277 (JP93), AB255289 (JP151)	Bamboo	–	Morakotkarn et al. (2007)
	Strain JP7, JP93, JP119, JP151, JP185, JP232, and JP256	AB354993 (JP119), AB354994 (JP185), AB354995 (JP256)	Bamboo	–	Morakotkarn et al. (2008)
	*Shiraia* sp. SUPER-H168	EU267793	*Brachystachyum densiflorum*	HA-2.02 mg/g dry solid substrate	Liang et al. (2009b)
	*Shiraia* sp. Slf14	GQ355934	*Huperzia serrata*	–	Zhu et al. (2010)
	*Shiraia* sp. Slf14	GQ355934	*H. serrata*	HA-HC, EA-EC	Tong et al. (2017)
	*Shiraia* sp. Slf14	GQ355934	*H. serrata*	PQ-305.066 mg/L	Liu et al. (2020)
	ZZZ-817	–	*Phyllostachys edulis*	HA-1,760.9 mg/L	Li et al. (2010)
	Strain MSX60519	MN970609	Dry leaf litter	Hypocrellins, *ent*-shiraiachrome A, hypomycin E	Al Subeh et al. (2020)
	*Shiraia* sp. A8	FJ560594	*B. densiflorum*	HA-110.04 mg/L	Zhang et al. (2014)
	NU_12_, UV_4_	–	*B. densiflorum*	HA-30.1 mg/L for NU_12_, 50.6 mg/L for UV_4_	Dong et al. (2012)
	*Shiraia* sp. strain ZZZ816	HQ696072	*P. edulis*	HA-921.6 mg/L	Shen et al. (2016)
Other endophytes	*Pseudoshiraia conidialis* CNUCC 1353PR	MZ519527	Bamboo	HA-677.11 mg/L, HB-155.36 mg/L, SA-152.31 mg/L, EA-326.59 mg/L, EB-60.41 mg/L, EC-38.36 mg/L	Tong et al. (2021)
	*Phaeosphaeria* sp.	HQ324780	*Heterodermia obscurata* (Nyl.)	Phaeosphaerins A-2.8 mg/4.0 g crude extract, Phaeosphaerins B-2.0 mg/4.0 g crude extract, Phaeosphaerins C-7.2 mg/4.0 gcrude extract, Phaeosphaerins D-6.8 mg/4.0 g crude extract, Phaeosphaerins E-1.6 mg/4.0 g crude extract, Phaeosphaerins F-1.8 mg/4.0 g crude extract, HA-15.7 mg/4.0 g crude extract, HC-1.2 mg/4.0 g crude extract, elsinochromes A-0.9 mg/4.0 g crude extract, elsinochromes B-12.0 mg/4.0 gcrude extract, elsinochromes C-6.7 mg/4.0 g crude extract, (+)-calphostin D-3.2 mg/4.0 g crude extract	Li et al. (2012)
	*Penicillium chrysogenum*	–	*Fagonia cretica*	HB-880.0 mg/1.5 g crude extract, HC-24.0 mg/1.5 g crude extract	Meng et al. (2011)

### 2.2. Hypocrellin-yielding endophytes

#### 2.2.1. Shiraia*-like endophytes*

Recently, endophytic fungi from plants have been widely accepted as an important source of bioactive metabolites. There is an abundanance of endophytic fungi in bamboo (Hyde et al. 2002). Notably, certain species of *Shiraia* have been identified as endophytes within bamboo tissues (Table 1, Figure 3). Morakotkarn et al. (2007) isolated 257 strains of endophytic fungi from Japanese bamboos (*Phyllostachys* and *Sasa*), of which 71 representative strains were characterised using the 18S rRNA gene and internal transcribed spacer (ITS) region sequencing. Three endophytic strains (g05, g74, and g43) exhibited similarities of 91%–94% to *Shiraia* sp. ML-2004, while strain g58 was closely related to *S. bambusicola*. Additionally, seven strains of *Shiraia*-like fungi were isolated from fresh bamboo nodes, internodes, and leaf tissues as endophytes closely related to *S. bambusicola* (Morakotkarn et al. 2008). Among these, group A *Shiraia*-like endophytes exhibited deeply red-pigmented mycelium, along with distinct prawn-shaped conidioma-like structures, setting them apart from *S. bambusicola*. Liang et al. (2009b) isolated 453 fungal strains from bamboo tissues (*Brachystachyum densiflorum*), among which *Shiraia* sp. SUPER-H168 was found to produce HA at 2.02 mg/g dry solid. *Shiraia* sp. Slf14 isolated from the leaves of *Huperzia serrata* is a novel huperzine A – producing fungus, which also produces HA, HB, and HC (Zhu et al. 2010; Tong et al. 2017). In a fermentation medium with glucose as the carbon source, the total perylenequinone production (HA, HB, and elsinochrome A–C) of *Shiraia* sp. Slf14 reached 305.066 mg/L (Liu et al. 2020). Li et al. (2010) obtained endophytic *S. bambusicola* yielding HA at 1,760.9 mg/L in liquid cultures. Ent-SA, hypocrellins, and hypomycin E were produced by *Shiraia* sp. MSX60159 (Al Subeh et al. 2020). Additionally, our group screened endophytic *Shiraia* spp. from bamboo culms of *B. densiflorum* (Zhang et al. 2014). *Shiraia* sp. A8 produced HA at 110.04 mg/L after 10 days of mycelium culture. Two mutant strains, NU_12_ and UN_4_, of endophytic *Shiraia* sp. S8, generated via UV and nitrosoguanidine mutagenesis, produced HA at 30.1 and 50.6 mg/L, respectively, in mycelium cultures (Dong et al. 2012). Moreover, Shen et al. (2016) screened 14 isolates of *Shiraia* endophytes from the moso bamboo (*Phyllostachys edulis*) seeds. The culture conditions of *Shiraia* sp. strain ZZZ816 under submerged fermentation were optimised, and a higher HA yield of 921.6 mg/L was obtained.

#### 2.2.2. Other endophytes

Recently, a novel species, *Pseudoshiraia conidialis* gen. et sp. nov. within the genus *Pseudoshiraia* of Shiraiaceae, was isolated from bamboo tissues and identified based on morphological characteristics and phylogenetic analysis (Tong et al. 2021). Notably, *P. conidialis* CNUCC 1353PR exhibited a higher yield of total perylenequinones (1,410.13 mg/L) and HA (677.11 mg/L). Furthermore, an endolichenic fungus, *Phaeosphaeria* sp., from the lichen *Heterodermia obscurata*, produced six novel perylenequinones, phaeosphaerins A–F, along with six known perylenequinones (HA, HC, elsinochromes A–C, and (+)-calphostin D) (Li et al. 2012). Additionally, HB and HC were obtained from the endophytic *Penicillium chrysogenum* isolated from the non-bamboo host *Fagonia cretica* (Meng et al. 2011). These findings indicate that hypocrellins can be produced by fungi not belonging to Shiraiaceae. Moreover, the high yield of hypocrellins from endophytic fungi presents a promising new source for the development of photodynamic therapy agents.

## 3. Mycelium cultures for production of hypocrellins

The escalating demand for hypocrellins in various applications necessitates their production on a large scale. However, the intricate structure of hypocrellins renders their total synthesis challenging (e.g. 19 steps with an overall yield of 1.6%) (O’Brien et al. 2010). Consequently, fruiting bodies remain the primary source for commercial supply of hypocrellins. However, as *S. bambusicola* is a causal agent of bamboo blight diseases, leading to significant degradation of bamboo forests, and artificial cultivation of the fungus has not been successful (Liu et al. 2012b), reliance solely on wild *Shiraia* fruiting bodies cannot meet the increasing demand for hypocrellins in widespread medical and industrial applications. Therefore, there is a pressing need to develop more reliable methods for production of hypocrellins. Recently, *Shiraia* mycelium cultures have emerged as a promising alternative. Methods for solid-state or submerged fermentation of *Shiraia* have been established, and culture conditions have been optimised, including the inoculum level, initial moisture content and pH, medium composition, and incubation time (Liang et al. 2009b; Yang et al. 2009; Cai et al. 2010; Dong et al. 2012).

### 3.1. Solid-state culture

In solid-state culture, *Shiraia* is typically cultivated on potato dextrose agar (PDA) plates or in conical flasks. The average diameter of a *Shiraia* colony on PDA plates, when incubated at 28 °C for 8 days, is approximately 8–10 cm (Figure 2(C,D)). In the PDA medium, three main types of hyphae are observed: biofilm, penetrative, and aerial hyphae (Gao et al. 2018b). Biofilm hyphae are those extending above the substrate surface to form biofilms during mycelial growth. The pigments secreted by biofilm hyphae impart a light or dark reddish colouration to both the surface and reverse side of the colony. As depicted in Figure 2(B), these pigments are presumed to be perylenequinones, as indicated by specific colour reactions: red in acid solution, dark purple with FeCl_3_, and green in alkaline solution (Yang et al. 2009). The composition of individual perylenequinones (HA-HC and EA-EC) is determined using HPLC (Tong et al. 2017). The primary perylenequinone component produced in solid-state culture by *Shiraia* is HA.

Various media are employed in *Shiraia* cultures for production of hypocrellins depending on medium composition, pH, temperature, light exposure, and other conditions (Table 2). PDA medium is commonly utilised for preserving *Shiraia* isolates. The solid media for the production of hypocrellins typically comprise grains, wheat bran, and other agricultural products, supplemented with a small amount of inorganic salts (Table 2). *Shiraia* fungi exhibit versatility in utilising various plant residues and carbon sources, with corn being identified as the optimal substrate and glucose as the preferred carbon source for HA production in *Shiraia* sp. SUPER-H168 (Cai et al. 2010). The type and concentration of nitrogen sources have varying impacts on hypocrellin production in solid-state culture. Organic nitrogen sources such as yeast extract and peptone have been observed to inhibit PQ pigment production, whereas inorganic nitrogen sources, including urea, NaNO_3_, and (NH_4_)_2_SO_4_, promote hypocrellin synthesis in *S. bambusicola* (Liang et al. 2009a; Cai et al. 2010). Following optimisation, the production of hypocrellins increased to 16.6 mg/gds (per gram of initial dry solids) in solid-state fermentation using substrates of corn and straw powder supplemented with glucose and NH_4_Cl (Lv et al. 2013). Additionally, the size of the inoculum (10^4^–10^6^ spores/g) has been identified as a crucial factor for HA production in solid-state culture of *Shiraia* sp. SUPER-H168 (Cai et al. 2010). An initial moisture content of 50% has been found to be optimal for fungal growth and HA production in solid-state culture (Liang et al. 2009b; Cai et al. 2010). Furthermore, a culture temperature range of 25–30 °C has been shown to promote the accumulation of hypocrellins.

**Table 2.** The culture medium and condition for *Shiraia* mycelium culture.

Medium components	Culture condition	PQ pigment yields	References
Solid-state culture			
Corn as substrate, glucose 1.65g/100 g, NaNO_3_ 0.43 g/L, K_2_HPO_4_ 1 g/L, KCl 0.5 g/L, MgSO_4_‧7H_2_O 0.5 g/L, FeSO_4_ 0.01 g/L	Incubated at dark, inoculum size 3 × 10^6^ spores, substrate particle size 0.8–1 mm, initial moisture content 50%, temperature 30 °C, incubation period 18 d	HA-4.7 mg/g	Cai et al. (2010)
Corn grits 833.3 g/L, wheat bran 166.7 g/L, glucose 50 g/L, NaNO_3_ 5 g/L, ZnSO_4_‧7H_2_O 1 g/L	Initial moisture content 50%, initial pH 7.0, temperature 30 °C, incubation period 18 d	Hypocrellins-9.37 mg/g dry solid	Liang et al. (2009a)
Maize 100 g/L, wheat straw 100 g/L, glucose 50 g/L, NH_4_Cl 10 g/L, CuSO_4_ 0.5 g/L, CaCl_2_ 1 g/L, KH_2_PO_4_ 0.5 g/L, K_2_HPO_4_ 1 g/L, MgSO_4_ 2 g/L	Initial moisture content 50%, temperature 30 °C, initial strain age 24 h, inoculation amount 2 mL/30 g dry solid, incubation period 15 d	Hypocrellins-16.6 mg/gds (per gram of initial dry solids)	Lv et al. (2013)
Rice 1,200 g/L, K_2_HPO_4_ 1 g/L, KCl 0.5 g/L, MgSO_4_‧7H_2_O 0.5 g/L, FeSO_4_ 0.01 g/L	Incubated at dark, inoculation amount 2 mL (1× 10^6^ spores/mL), initial pH 7.5, moisture content 50%, temperature 30 °C, incubation period 15 d	HA-2.02 mg/g dry solid	Liang et al. (2009b)
Potato 200 g/L, glucose 20 g/L	Incubated at dark for 10 d, temperature 30 °C	Hypocrellins-13.73 mg per dish	Gao et al. (2018b)
Liquid mycelium culture			
Glucose 45.7 g/L, (NH_4_)_2_SO_4_ 1.93 g/L, K_2_HPO_4_ 1.0 g/L, MgSO_4_‧7H_2_O 0.5 g/L, KCl 0.5 g/L	Incubated at 175 r/min for 5 d, temperature 25 °C	Hypocrellins-196.94 mg/L	Yang et al. (2009)
Potato extract 200 g/L, yeast extract 5 g/L, fructose 60 g/L	Incubated at 150 r/min for 14 d, temperature 28 °C, medium amount 140 mL/500 mL (v/v)	PQ-1,753.64 mg/L	Liu et al. (2020)
potato extracts 200 g/L, glucose 30 g/L, KH_2_PO_4_ 2 g/L, MgSO_4_‧7H_2_O 0.5 g/L	Incubated at 120 r/min for 168 h, temperature 26 °C, pH 5.5–6.0, medium amount 50 mL/500 mL (v/v)	Hypocrellins-45 mg/L	Shi et al. (2004)
Yeast extract 20 g/L, malt sugar 40 g/L, FeSO_4_‧H_2_O 0.5 g/L, urea 4.0 g/L, MgSO_4_‧7H_2_O 0.5 g/L	Incubated at 130 r/min for 144 h, initial pH 6.0, mycelial age 60 h, inoculation level 10%, temperature 25 °C, medium amount 100 mL/500 mL (v/v)	HA-921.6 mg/L	Shen et al. (2016)
Glucose 20 g/L, NaNO_3_ 2 g/L, KH_2_PO_4_ 1 g/L, MgSO_4_ 0.5 g/L	Initial pH 7.5	Hypocrellins-28.04 mg/g (dry weight)	Xiang (2010)
Potato extract 200 g/L, fructose 60 g/L, L-arginine 7 g/L	Temperature 28 °C, incubation period 14 d	PQ-2,424.34 mg/L in strain Slf14, PQ-817.64 mg/L in strain Slf14 (w)	Chen et al. (2022)
Potato extract 200 g/L, glucose 20 g/L, KH_2_PO_4_ 1 g/L, MgSO_4_ 0.5 g/L, KCl 0.5 g/L, FeSO_4_‧7H_2_O 0.01 g/L, yeast extract 3 g/L, peptone 10 g/L, L-valine 1.5 g/L	Incubated at 150 r/min for 8 d, temperature 28 °C, medium amount 50 mL/150 mL (v/v)	HA-237.92 mg/L	Shen et al. (2023a)
Potato extract 200 g/L, glucose 20 g/L, KH_2_PO_4_ 3 g/L, MgSO_4_ 1.5 g/L, VB_1_ 0.01 g/L, yeast extract 5 g/L	Incubated at 150 r/min for 8 d, temperature 28 °C	HA-10–20 mg/L	Pan et al. (2012)
Potato extract 100 g/L, starch 20 g/L, NaNO_3_ 4 g/L, KH_2_PO_4_ 1.5 g/L, CaCO_3_ 0.5 g/L, VB_1_ 0.01 g/L, SNP 5 μmol/L	Incubated at 200 r/min for 8 d, initial pH 6.3, temperature 28 °C, medium amount 50 mL/150 mL (v/v), red light (627 nm) at 200 lx	HA-254 mg/L	Wang et al. (2024)
Glucose 47.33 g/L, (NH_4_)_2_SO_4_ 2.14 g/L, KH_2_PO_4_ 2.87 g/L, MgSO_4_ 1.68 g/L, soybean oil 10 g/L	Incubated at 180 r/min for 5 d, temperature 25 °C, medium amount 100 mL/500 mL (v/v)	257.66 mg/L	Bu and Yang (2020)

### 3.2. Liquid mycelium culture

Compared to solid-state fermentation, submerged liquid culture offers advantages such as scalability, higher yield, and shorter culture time. *Shiraia* mycelium can be cultivated in submerged liquid culture using a wide range of carbon and nitrogen sources (Table 2). Various carbon sources, including glucose, fructose, sucrose, xylose, maltose, or starch, have been investigated for their impact on the production of hypocrellins (Yang et al. 2009; Bu and Yang 2020; Liu et al. 2020). For instance, when glucose at 30 g/L was used as the carbon source, *S. bambusicola* LBR-SB exhibited higher biomass (32.5 g/L) and hypocrellin production (26.1 mg/L) (Shi et al. 2004). Liu et al. (2020) reported that fructose at 60 g/L favoured total perylenequinone production of 1,753.64 mg/L by endophytic *Shiraia* sp. Slf14, followed by sucrose, maltose, and glucose. Moreover, other carbon sources like xylose and maltose were found to be suitable for HA production in submerged liquid culture of *Shiraia* sp. strain ZZZ816 (Shen et al. 2016). These findings suggest that different carbon sources influence the growth of *Shiraia* strains and the biosynthesis of individual perylenequinones in liquid cultures. Similarly, the choice of nitrogen source significantly affects *Shiraia* hypocrellin production. Generally, organic nitrogen sources such as yeast extract, peptone, and beef extracts are more conducive to hypocrellin biosynthesis than inorganic nitrogen sources like sodium nitrate or ammonium nitrate (Liang et al. 2009a; Shen et al. 2016; Liu et al. 2020). However, Xiang (2010) suggested that urea or NaNO_3_ was the optimal nitrogen source for hypocrellin production after optimising the cultural conditions of *S. bambusicola*. Additionally, certain amino acids such as arginine and phenylalanine were found to enhance perylenequinone production in *Shiraia* sp. Slf14(w) (Chen et al. 2022). Notably, branched-chain amino acids (BCAAs) exhibited contrasting effects on *Shiraia* growth and perylenequinone production. Specifically, PQ production (HA, HC, and EA-EC) was significantly stimulated by l-isoleucine (l-Ile) and l-valine (l-Val), while being sharply inhibited by l-leucine (l-Leu) (Shen et al. 2023a). These findings highlight the role of nitrogen source metabolism in *Shiraia* hypocrellin biosynthesis. Optimum concentrations of media components for hypocrellin production were determined to be (w/v): 20% potato powder, 2% glucose, 0.1% KH_2_PO_4_, 0.05% MgSO_4_, 0.05% KCl, 0.001% FeSO_4_·7H_2_O, 0.3% yeast extract, and 1% peptone (pH 6.5) (Shen et al. 2023a).

**Figure 4.**
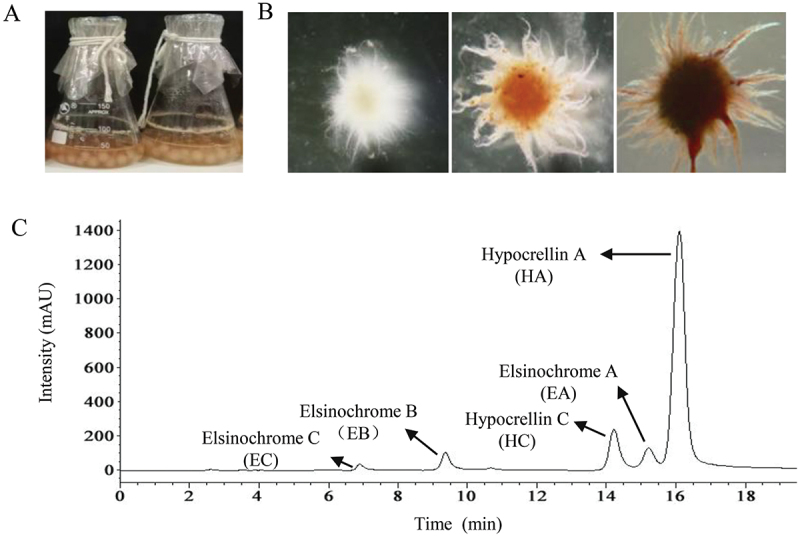
The fungal pellet formation and perylenequinone production in liquid culture of *Shiraia bambusicola*. (A) The culture was maintained in 150-mL flask containing 50 mL of liquid medium at 150 r/min and 28 °C. (B) Morphology (100 ×) of the pellet during the cultivation. (C) The chromatogram of individual perylenequinone in the mycelium. The figure was redrawn based on findings from our previous study (Wang et al. 2024).

In the liquid culture of *Shiraia*, the growth typically follows a pattern where a lag phase of 1–2 days is observed, followed by entry into the exponential growth phase (day 3–5). Subsequently, there is a significant accumulation of perylenequinone pigments after 6–9 days, marking the stationary phase. Generally, the liquid-state culture of *Shiraia* sp. S8 lasts from 8 to 10 days, with HA production ranging from 10 to 20 mg/L (Pan et al. 2012). During the liquid culture (Figure 4), smaller and roundish pellets begin to appear after 12 h, with shorter hyphae extending out of the pellets. The content of individual perylenequinone such as HA, HC, and EA-EC is detected in the mycelia of *Shiraia* sp. S9 (Wang et al. 2024).

## 4. Elicitation of HA production

Given the relatively lower content of hypocrellins, such as 2.02 mg/g dry weight for HA in solid-state fermentation (Liang et al. 2009b) or approximately 10–40 mg/L in liquid fermentation (Liu et al. 2009), it becomes imperative to address the bottleneck of lower production of hypocrellins for medical applications. One of the most effective strategies for enhancing fungal secondary metabolite production is elicitation (Bharatiya et al. 2021). Elicitors are primarily categorised into two types: biotic elicitors and abiotic elicitors, based on their origin and nature. Abiotic elicitors encompass environmental factors applied to fungal cultures to induce various physiological processes and the biosynthesis of fungal secondary metabolites, including light exposure, salinity, low/high temperature, and heavy metals. Biotic elicitors, on the other hand, are living organisms or substances of biological origin, such as proteins, carbohydrates, and crude extracts, which can activate the accumulation of fungal secondary metabolites. Table 3 presents the elicitors used to stimulate the production of hypocrellins.

**Table 3.** The elicitors for the improved production of hypocrellins in *Shiraia* mycelium cultures.

Elicitor type	Elicitor agents	Pigment yields and elicitor folds	References
Biotic elicitors			
Live bacterial cells	*Pseudomonas putida*, *P. fulva*, *P. parafulva*	HA-3.77–4.01 mg/cm^2^ for *P. putida* (1.27–1.35-fold), 4.07–6.18 mg/cm^2^ for *P. fulva* (1.38–2.09-fold), 4.15 mg/cm^2^ for *P. parafulva* (1.40-fold)	Ma et al. (2019a)
	*P. fulva* SB1 (400 cells/mL)	HA-325.87 mg/L (3.20-fold)	Ma et al. (2019b)
Bacterial polysaccharides	Exopolysaccharide of SB1 (30 mg/L)	HA-349.51 mg/L (3.33-fold)	Zhou et al. (2023)
	Lipopolysaccharide of SB1 (20 μg/mL)	HA-303.76 mg/L (2.19-fold)	Li et al. (2024)
Bacterial volatiles	Bacterial volatiles of *Bacillus cereus* No.1	HA-225.9 mg/L (1.87-fold)	Xu et al. (2022)
Fungal elicitors	TT1 (81.40 μg/mL)	Hypocrellins-102.6 mg/L (7.9-fold)	Du et al. (2013)
	Crude polysaccharides of TT1 (50 μg/mL)	Hypocrellins-23.89 mg/L (80% increase)	Du et al. (2014)
	Mixed extracts of *Aspergillum niger* (50 μg/mL)	Hypocrellins-90 mg/L (6.2-fold)	Du et al. (2015)
	*Arthrinium* sp. AF-5 (0.06 g FW/mL)	HA-667.47 mg/L (4-fold)	Yan et al. (2021)
	PB90-Protein of *Phytophthora boehmeriae* (5 nmol/L)	Hypocrellins-278.71 mg/L (4.5-fold)	Du et al. (2019)
Plant elicitor	Bamboo charcoal powder (2 g/L)	HA-604.81 mg/L (1.6-fold)	Li et al. (2019)
	Bamboo polysaccharide BPSE (10 mg/L)	HA-422.8 mg/L (4.0-fold)	Shen et al. (2023b)
Abiotic elicitors			
Surfactant	Triton X-100 (0.6%)	Hypocrellins-780.6 mg/L	Cai et al. (2011)
	Triton X-100 (2.5%)	HA-96.9 mg/L	Lei et al. (2017)
	Triton X-100 (25 g/L)	HA-206.2 mg/L (5.4-fold)	Li et al. (2020)
Ultrasound	40 kHz, 0.28 W/cm^2^	HA-247.67 mg/L (3-fold)	Sun et al. (2017)
Light radiation	Light/dark shift (24/24 h, 200 lx)	HA-181.67 mg/L (73% increase)	Sun et al. (2018)
	Continuous LED light	–	Al Subeh et al. (2020)
	Red light (200 lx)	HA-175.53 mg/L (3.8-fold)	Ma et al. (2019)
	Blue light (6 h/day, 200 lx)	HA-242.76 mg/L (2.27-fold)	Li et al. (2022)
Temperature	26 °C	PQ-0.41 chromo value (6.3-fold)	Li et al. (2003)
	26 °C	Hypocrellins-40 mg/kg	Cai et al. (2004)
	28 °C	Hypocrellins-2.7 mg/g	Hu et al. (2008)
	32 °C	HA-(400%–600% increase)	Wen et al. (2022)
	40 °C	PQ-577 mg/L (20.89-fold)	Xu et al. (2023)
Heavy metal ions	Ca^2+^ (CaCl_2_, 6.0 g/L)	PQ-1,894.66 mg/L (5.8-fold)	Liu et al. (2018)
	La^3+^ (LaCl_3_, 1.0 g/L)	HA-225.05 mg/L (1.56-fold)	Lu et al. (2019)
Signal molecules	NO (SNP-0.01 mmol/L)	PQ-(156% increase)	Zhao et al. (2021)
	NO (SNP-0.02 mmol/L)	HA-110.34 mg/L (2.65-fold)	Ma et al. (2021)
	NO (SNP-0.1 mmol/L), SA (1 mmol/L)	Hypocrellins-118 mg/L (5-fold)	Du et al. (2015)
	H_2_O_2_ (10/20 mmol/L)	Hypocrellins-1,000 mg/L (25%–27% increase)	Deng et al. (2016)
	H_2_O_2_ (10 μmol/L)	–	Lu et al. (2019)
	H_2_O_2_ (30 μmol/L)	HA-256.6 mg/L (2.5-fold)	Zhang et al. (2014)

SB1-*Pseudomonas fulva* SB1; TT1-*Trametes* sp. GZUIFR-TT1; PB90-*Phytophthora boehmeriae*.

### 4.1. Biotic elicitors

#### 4.1.1. *Bacteria from* Shiraia *fruiting bodies*

**Figure 5.**
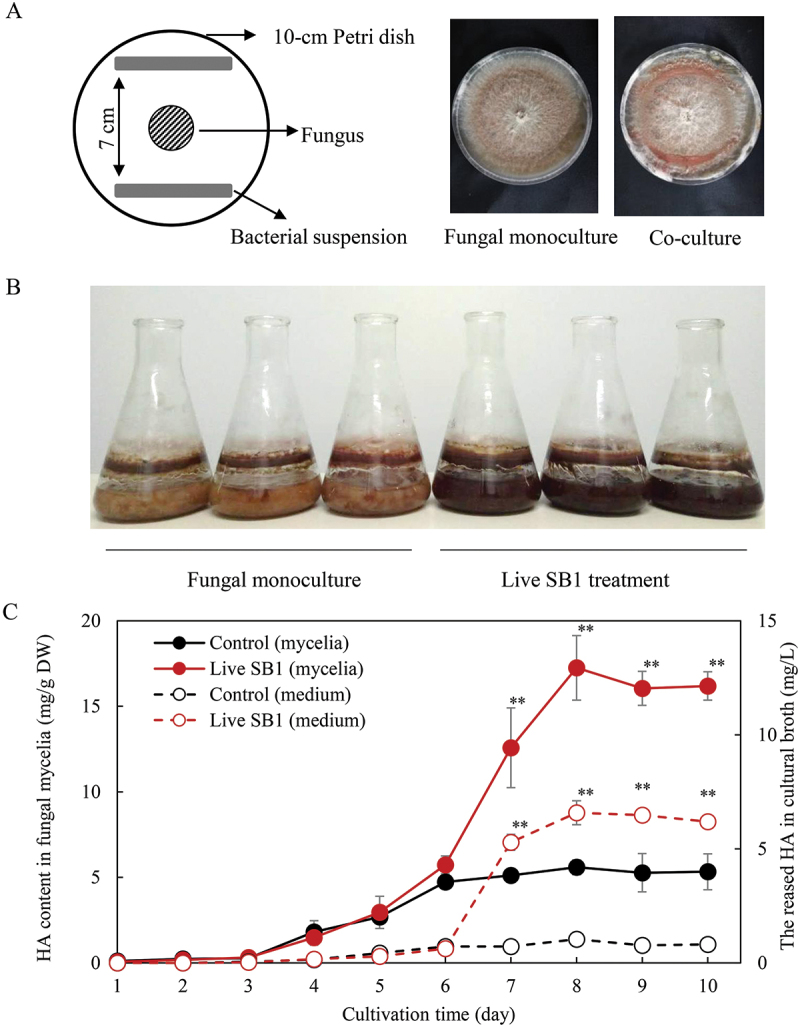
The effects of live *Pseudomonas fulva* SB1 on the growth and hypocrellins production of *Shiraia* sp. S9. (A) Scheme of the *in vitro* confrontation assay. A small piece (5 mm × 5 mm) of the fungal strain was placed in the center of 10-cm PDA plate at 28 °C for 4 d. The bacterial suspension (10 μL) was streaked in two parallel straight lines on PDA, approximately 7 cm apart from each other. (B) The liquid submerged culture of *Shiraia* sp. S9 with or without live SB1 treatment at 400 cells/mL on day 6. (C) Time profiles of HA content in mycelium and the released HA in cultural broth in the submerged culture. Values are mean ± SD from three independent experiments (**p < 0.01 versus control). The figure was redrawn based on findings from our previous study (Ma et al. 2019a, 2019b).

Many studies have indicated that fungal fruiting bodies harbour a diverse bacterial community (Carrasco and Preston 2020). These bacteria have been found to exert both positive and negative effects on mycelial growth, spore germination, and fruiting body formation (Oh et al. 2018). Utilizing high-throughput sequencing, we identified a rich bacterial community within *Shiraia* fruiting bodies, comprising 723 bacterial operational taxonomic units (OTUs) belonging to 30 bacterial phyla, 84 classes, 149 orders, 244 families, and 364 genera. The most abundant bacterial OTUs were assigned to *Bacillus* (10.86%) and *Pseudomonas* (4.37%) (Ma et al. 2019a). Furthermore, we isolated 31 bacterial strains from *Shiraia* fruiting bodies using a culture-dependent method. Through fungus-bacteria confrontation assays (Figure 5(A)), we observed that six isolates from *Pseudomonas*, including *P. putida*, *P. fulva*, and *P. parafulva*, could stimulate PQ accumulation in *Shiraia* sp. S9. Conversely, five *Bacillus* isolates completely suppressed fungal PQ production. Specifically, the individual PQ (HA, HC, EA, and EB) content was significantly stimulated by treatment with live *P. fulva* SB1 (Figure 5(B,C)). Application of the bacterium *P. fulva* SB1 at 400 cells/mL to the mycelium cultures of *Shiraia* sp. S9 on day 6 not only enhanced the HA content within the hyphae but also increased the secreted HA in the medium, resulting in the highest HA production (325.87 mg/L) on day 8, approximately 3.20-fold higher than that observed in axenic culture (Ma et al. 2019b).

#### 4.1.2. Bacterial EPS and LPS

**Figure 6.**
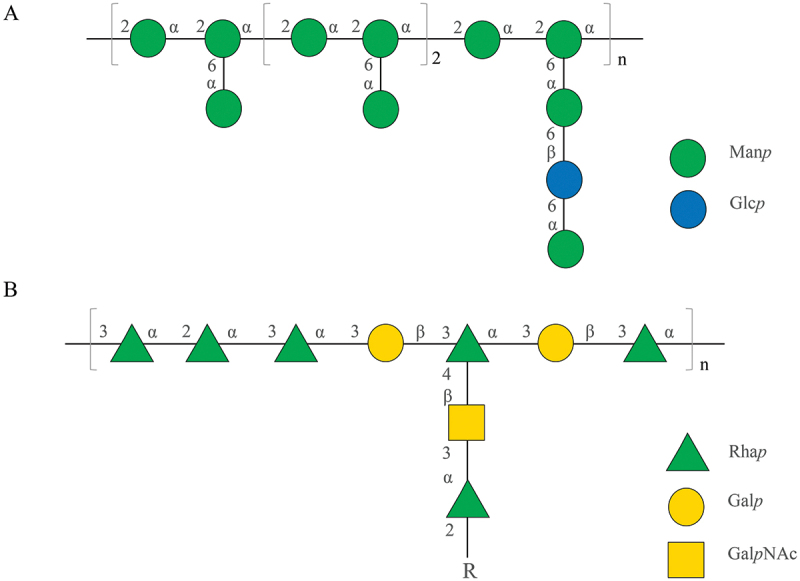
The proposed structure of EPS-1 (*n* ≈ 37) (A) and the repeating unit of OPS (*n* ≈ 1,600; *R* = t-Rha*p*, t-Gal*p*) (B). The figure was redrawn based on findings from our previous study (Zhou et al. 2023; Li et al. 2024).

Extracellular polysaccharides (EPS) serve as major virulence factors in bacterial pathogens such as *P. solanacearum* and *Ralstonia solanacearum*, contributing to wilt in tomato plants (Denny and Baek 1991). Interestingly, microbial EPS have been found to induce plant secondary metabolites, including flavonoid production in *Fagopyrum tataricum* (Zhao et al. 2015), diosgenin production in *Dioscorea zingiberensis* (Li et al. 2011), and volatile oils in *Atractylodes lancea* (Chen et al. 2016). We isolated crude EPS from *P. fulva* SB1 by precipitating bacterial culture broth with graded ethanol (40%–85% v/v) (Zhou et al. 2023). Most EPS fractions were found to enhance fungal HA production. The active EPS, designated EPS-1, was separated using DEAE-FF Sepharose and Sephadex G-100 columns. EPS-1, identified as a mannan-rich branched heteropolysaccharide consisting of mannose (Man) and glucose (Glc) with an average molecular weight of 9.213 × 10^4^ Da (Figure 6(A)), increased fungal HA production in mycelium culture to 349.51 mg/L at a concentration of 30 mg/L, over 3.0-fold compared to the control. The stimulating effects of EPS-1 were attributed to the activation of transcriptional levels of hypocrellin biosynthetic genes and transporters. Furthermore, we observed colonisation of mature ascospores’ surfaces by bacteria in fruiting bodies and extensive bacterial colonisation of fungal hyphae during bacterial-fungal co-culture (Ma et al. 2019a, 2019b). In direct contact between bacteria and other cells, lipopolysaccharides (LPS) from bacterial surfaces act as the primary active agents (Kutschera and Ranf 2019). LPS from *Escherichia coli* O55:B5, *Salmonella typhi* O901, *Pseudomonas aeruginosa* 10 (Ps-LPS), and *P. fulva* SB1 at 20 μg/mL significantly enhanced fungal HA contents in *Shiraia* sp. S9 (Li et al. 2024). Removal of LPS from *P. fulva* SB1 cell walls abolished the enhanced HA production, indicating the eliciting role of LPS during direct contact with *Shiraia* sp. S9. The bacterial LPS was purified, and the *O*-specific polysaccharide (OPS) was characterised as a branched heteropolysaccharide consisting of rhamnose, galactose, and N-acetyl-galactosamine with an average molecular weight of 282.8 kDa (Figure 6(B)). LPS induced nitric oxide (NO) generation to elicit fungal HA production by upregulating the expressions of critical genes for central carbon metabolism and HA biosynthesis. Treatment with *P. fulva* SB1 LPS at 20 μg/mL on day 3 increased fungal HA production to 303.76 mg/L in an 8-day culture of *Shiraia* sp. S9, approximately 2.19-fold over the control group (Li et al. 2024).

#### 4.1.3. Bacterial volatiles

**Figure 7.**
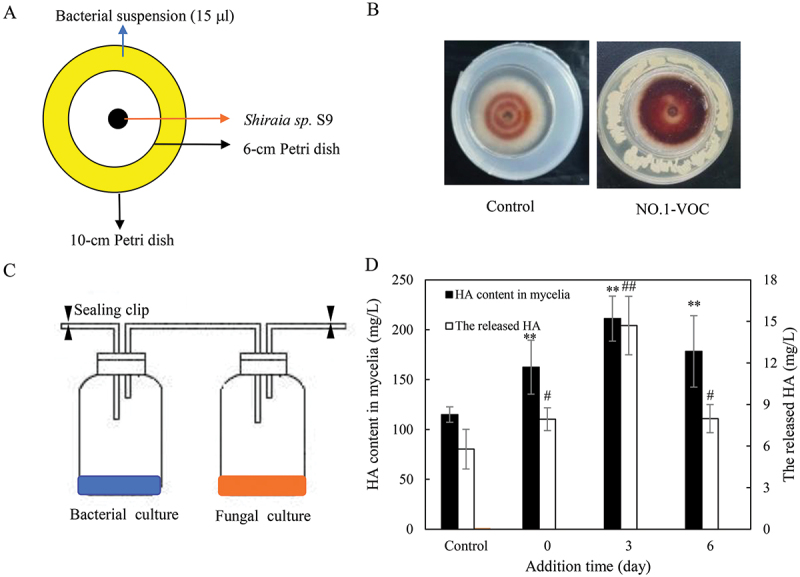
Effects of bacterial volatiles of *Bacillus cereus* No.1 on *Shiraia* HA production in the submerged volatile co-cultures. The mode diagrams (A) and the “donut” plate (B) for the bacterial volatiles and the fungus *Shiraia* sp. S9. The mode diagrams for the submerged volatile co-cultures (C). Two culture flasks were connected through sealed glass tube. The culture was maintained in 250-mL flask containing 100 mL of the liquid medium at 150 r/min and 28 °C for 8 d. An equal volume of sterile LB broth instead of bacterial suspension added to flask was used as control group. Effect of addition time for the bacteria on fungal HA content in mycelium or in medium during the submerged volatile co-cultures (D). Values are mean ± SD from three independent experiments. Different letters above the bars mean significant differences (**, ^##^*p* < 0.01 versus control, *, ^#^*p* < 0.05). The figure was redrawn based on findings from our previous study (Xu et al. 2022).

Bacterial volatiles have been found to significantly impact plant growth and exhibit strong inhibitory activity against plant pathogens (Zhang et al. 2019). Recently, volatile organic compounds (VOCs) produced by certain bacteria have been shown to alter fungal metabolism, such as suppressing pigment accumulation in *Fusarium oxysporum* and reducing sclerotia biosynthesis in *Sclerotinia sclerotiorum* (Massawe et al. 2018). In our previous studies, although 14 isolates of dominant *Bacillus* exhibited various degrees of suppression of fungal production of hypocrellins in confrontation tests (Ma et al. 2019a), volatiles produced by *B. cereus* No.1 were found to promote production of hypocrellins in the fungus *Shiraia* sp. S9 in plate assays using a “donut” plate assay (Figure 7(A,B)) (Xu et al. 2022). We established a submerged volatile co-culture for eliciting bacterial volatiles on fungal HA production (Figure 7(C)). When a flask containing bacterial suspension at 500 cells/mL was connected to a fungal culture on day 3, both mycelial content and released HA were enhanced, resulting in a total HA production of 225.9 mg/L, approximately 1.87 times that of the control group (Figure 7(D)). We identified 34 VOCs produced by *B. cereus* No.1 using GC-MS, and the eliciting compounds were phenylacetaldehyde, dimethyl disulphide, phenylethyl alcohol, hexadecane, and benzaldehyde (Xu et al. 2022).

#### 4.1.4. Fungal elicitors

Various components derived from fungal cells, such as polysaccharides, proteins, or mycelial homogenates, as well as culture filtrates, have been utilised as fungal elicitors to stimulate the biosynthesis of plant secondary metabolites (Zhao et al. 2005). PB90 is a protein elicitor with a molecular weight of 90 kDa isolated from *Phytophthora boehmeriae*, which was applied to cultures of *S. bambusicola* BZ-16X1 to promote hypocrellin production (Du et al. 2019). After 9 days of PB90 treatment at 5 nmol/L, hypocrellin production increased to 278.71 mg/L, about 2.5–4.5 times higher than that of the control. Du et al. (2013) isolated 11 species of endophytic fungi from bamboos, and autoclaved mycelial homogenate from *Trametes* sp. GZUIFRTT1 was found to stimulate hypocrellin production, referred to as fungal elicitor TT1. The addition of TT1 (81.40 μg/mL) on the third day of mycelial culture resulted in hypocrellin production of 102.60 mg/L, approximately 7.90 times higher than that of the control. Crude polysaccharides were further isolated and added at 50 μg/mL to 3-day-old cultures of *S. bambusicola* GZUIFR-08K1 (Du et al. 2014). Hypocrellin yield increased to 23.89 mg/L, an 80% increase over the control. Autoclaved mycelial homogenate from *Aspergillus niger* GZUIFR-S1 was applied at 50 μg/mL to the mycelium culture of *S. bambusicola* GZUIFR-11K1 to enhance hypocrellin production to 90 mg/L, approximately 6.2-fold higher than that of the control (Du et al. 2015). Yan et al. (2021) isolated 17 endophytic fungi strains from bamboo (*P. amarus*) and established a co-culture with *S. bambusicola* GDMCC 60438. When the endophytic *Arthrinium* sp. AF-5 was added at 0.06 g fresh weight (FW)/mL to the 2-day-old *Shiraia* culture, the yield of HA reached 667.47 mg/L after an 84 h co-cultivation, approximately 4 times higher compared to that in the mono-culture of *S. bambusicola*.

#### 4.1.5. Bamboo charcoal powder and polysaccharides

Recently, microparticles, including talc, Al_2_O_3_, and TiSiO_4_ particles, have been utilised in mycelium culture to control filamentous fungal growth for enhanced metabolite production (Karahalil et al. 2019). The addition of bamboo charcoal powder with a diameter of Ø = 2.3–5.5 μm to the preculture decreased the fungal pellet diameter of *S. bambusicola* and improved hypocrellin production by increasing the consumption of oxygen and sugar and up-regulating the gene expressions for HA biosynthesis (Li et al. 2019b). Bamboo charcoal powder at a concentration of 2.0 g/L increased HA contents both in mycelia by 44.9%–265.5% and in the medium by 57.0%–160.5%. Additionally, a bamboo polysaccharide with a molecular weight of 34.2 kDa was isolated with effective eliciting activity on hypocrellin biosynthesis (Shen et al. 2023b). After 5 days of bamboo polysaccharide treatment (at 10 mg/L), HA production in mycelium cultures of *Shiraia* sp. S9 increased to 422.8 mg/L, approximately 4.0 times that of the control.

### 4.2. Abiotic elicitors

#### 4.2.1. Surfactant treatment

**Figure 8.**
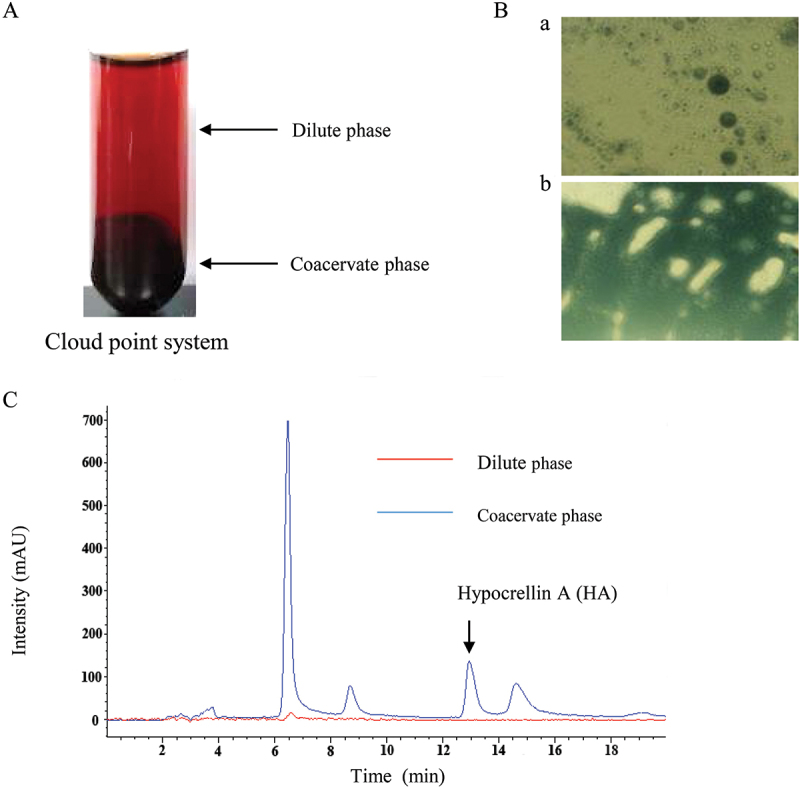
Distribution of hypocrellins in cloud point system. (A) Phase separation of Triton X-100 micelle aqueous solution. (B) Microscopic observation of the cloud point system stained with the oil soluble dye Sudan black B; a) dilute phase, oil-in-water emulsion (40×); b) coacervate phase, water-in-oil emulsion (40×). (C) The chromatogram of HA in cloud point system. The figure was redrawn based on findings from our previous study (Li et al. 2020).

Adding surfactants is a simple and effective strategy for stimulating the secretion of fungal secondary metabolites by modifying the cell membrane structure (Hu et al. 2012). In the mycelium culture of *Shiraia* sp. SUPERH168, the non-ionic surfactant Triton X-100 at concentrations ranging from 0.2% to 1.0% (w/v) was used as a component of the medium to induce biosynthesis of hypocrellins (Cai et al. 2011). In a previous study, no HA production was observed from mycelium or in the medium during the submerged culture of *S. bambusicola* S8. Eight surfactants, including Pluronic F68, Pluronic F-127, Tween-40, Tween-80, SDS, Brij 52, Span 80, and Triton X-100, were screened for their ability to induce HA production (Lei et al. 2017). Only Triton X-100 was found to have the induction ability. After Triton X-100 was added at a concentration of 2.5% (w/v) after 36 h of mycelial culture, both the biosynthesis of HA in the mycelium and the release of HA into the medium were stimulated, resulting in a total production of HA of 96.9 mg/L on day 8. Transcriptomic analysis showed that Triton X-100 treatment changed the expression of genes involved in transmembrane transport and biosynthesis of hypocrellins, indicating the eliciting role of Triton X-100 on HA biosynthesis and exudation. Furthermore, a two-phase system comprising an aqueous surfactant micelle solution in the upper layer (dilute phase) and a surfactant-rich lower layer (coacervate phase) was employed for extractive fermentation (Li et al. 2020). The extracellular broth of the culture under Triton X-100 treatment was further collected for cloud point extraction after the mycelia were harvested on day 8 (Figure 8(A)). After phase separation in the cloud point system at 75 °C, the extracellular HA was partitioned mainly into the coacervate phase (Triton X-100-rich phase) (Figure 8(B)). In the extractive *Shiraia* fermentation, total HA production reached 206.2 mg/L after 9 days, about 5.4 times that of the control (Figure 8(C)).

#### 4.2.2. Ultrasound

Ultrasound is another effective abiotic elicitor for stimulating secondary metabolite production in plant cells or mycelium cultures (Liu et al. 2012a; Lu et al. 2020). A low-intensity ultrasound (US) at 0.28 W/cm^2^ and 40 kHz frequency was applied thrice with repeated exposure durations of 5 min and intervals of 12 h to stimulate HA production in *S. bambusicola* cultures. This ultrasound exposure led to several observable effects, including decreased pellet diameter, fluffier pellets, enhanced membrane permeability, and alterations in the fatty acid composition of *S. bambusicola*. Furthermore, ultrasound exposure induced the generation of reactive oxygen species (ROS) and up-regulated the expression of genes related to HA biosynthesis and release, such as the polyketide synthase gene (*PKS*), *O*-methyltransferase/FAD-dependent monooxygenase (*Mono*), FAD/FMN-dependent oxidoreductase gene (*FAD*), and major facilitator superfamily transporter gene (*MFS*). As a result of ultrasound treatment, both the content of HA in mycelia and its release into the medium were increased. The total production of HA reached 247.67 mg/L, which was three times higher than that of the control (Sun et al. 2017).

#### 4.2.3. Light

**Figure 9.**
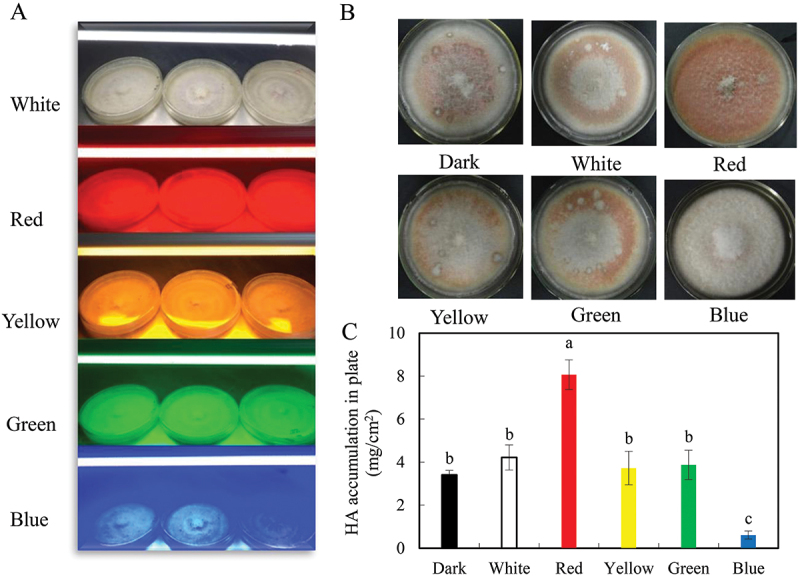
Effect of different wavelengths of light on fungal hypocrellin a (HA) production of *Shiraia bambusicola* S8 in solid-state cultures. (A) Fungus in PDA plate was kept at 28 °C for 8 d under different light treatments with LED lamps at 100 lx. (B) Fungal colony morphology in solid-state cultures under different light treatments. (C) HA content in solid state culture. Values are mean ± SD from three independent experiments. Different letters above the bars mean significant differences (*p* < 0.05). The figure was redrawn based on findings from our previous study (Ma et al. 2019).

Light plays a crucial role as an environmental signal for fungal metabolite production. Studies have shown varied effects of light on the growth and metabolite production of *Shiraia* spp. For instance, Gao et al. (2018b) found that light at 0.16 mW/cm^2^ promoted the growth of aerial hyphae in *Shiraia* sp. SUPER-H168 but suppressed accumulation of hypocrellins compared to dark conditions on solid plates. However, Sun et al. (2018) observed that high-intensity light at 600−800 lx inhibited both fungal growth and HA production in *S. bambusicola*, while lower intensity light at 200−400 lx increased HA production. Moreover, light/dark shifts have been investigated in mycelium cultures of *S. bambusicola*, revealing that a light/dark cycle of 24:24 h at 200 lx increased HA content in mycelia compared to dark conditions. Al Subeh et al. (2020) reported that light exposure facilitated the biosynthesis of hypocrellins and hypomycins in *Shiraia* sp. MSX60159, with continuous LED light exposure enhancing the production of these perylenequinone compounds. Furthermore, the influence of different light wavelengths on fungal HA production has been studied (Ma et al. 2019). While there was no significant difference in mycelium morphology, fungal biomass, and HA accumulation between dark control and white, yellow, or green light treatments at 100 lx (Figure 9), red light exposure resulted in intense red pigmentation and higher HA content in the medium. Transcriptomic analysis revealed that red light treatment altered gene expressions related to HA biosynthesis and transmembrane activity (Wang et al. 2024). Red light exposure at 200 lx increased HA yield significantly, with NO generation induced in *Shiraia* mycelia. The red light-induced NO regulated fungal HA biosynthesis through the NO-cGMP-PKG pathway. When the *Shiraia* mycelium culture was treated with the combined elicitation of red light with NO donor sodium nitroprusside (SNP) at 5 μmol/L, a higher level of HA at 254 mg/L was obtained, about 3.0-fold over the dark control. Interestingly, although longer exposure to blue light (8–24 h/day) at 150 lx or shorter treatment (6 h/day) at 300–400 lx suppressed HA content in the mycelia, the intermittent blue light (6 h/day) at 200 lx stimulated HA production significantly without any retardation of fungal growth (Li et al. 2022). When mycelium cultures were exposed to intermittent blue light at 470 nm for 8 d, HA production significantly increased compared to dark conditions. These findings demonstrate the complex and nuanced effects of light conditions on the fungal production of hypocrellins in *Shiraia* spp.

#### 4.2.4. Temperature stress

Although temperature stress can greatly reduce fungal growth and development, temperature changes (heat or cold stress) have also been shown to increase fungal secondary metabolite production (Brakhage 2013). In solid-state fermentation, Li et al. (2003) and Cai et al. (2004) found that an optimum temperature of 26 °C resulted in the highest yield of perylenequinones, with hypocrellin yields reaching approximately 40 mg/kg. Similarly, in the liquid culture of *S. bambusicola* ZH-5-1, an increase in temperature from 19 °C to 28 °C led to enhanced hypocrellin content in mycelia, reaching 2.7 mg/g (Hu et al. 2008). Wen et al. (2022) compared HA yields in the submerged cultivation of *S. bambusicola* GDMCC 60438 at different temperatures and found that the mycelial HA content was significantly promoted at 32 °C compared to 28 °C and 26 °C. This enhancement in HA production was attributed to the up-regulation of transcription factors and biosynthetic genes induced by high temperature, as revealed by RNA sequencing analysis. Furthermore, Xu et al. (2023) applied heat stress (HS) at 40 °C for 0–16 h on 2-day-old culture of *Shiraia* sp. Slf14(w) and then returned to 28 °C shaker cultures until day 8. They observed a significant increase in perylenequinone contents in fungal mycelium and medium. After 8 h of HS treatment, the total perylenequinone production reached 577 ± 34.56 mg/L on day 5, which was 20.89-fold improvement over the control. These findings highlight the potential of temperature stress as a convenient and effective elicitor for enhancing *Shiraia* perylenequinone production.

## 5. Conclusions and future prospects

Due to their exceptional photosensitisation properties and notable light-induced biological activities including antiviral, antileishmanial, antimalarial, and antimicrobial properties, hypocrellins have garnered significant interest as potential candidates for photodynamic therapy (PDT). Particularly, compounds such as HA, HB, and shiraiachrome A have shown promise as inhibitors of the severe acute respiratory syndrome coronavirus 2 (SARS-CoV-2), suggesting potential applications in the treatment of the novel coronavirus disease-2019 (COVID-19) (Law et al. 2021; Li et al. 2021). Presently, hypocrellins find widespread use as clinical PDT agents, as well as in applications such as food dyes and pesticides. The broad spectrum of potential applications has prompted increased attention towards fungal resources and biotechnological methods for the production of hypocrellins.

*Shiraia bambusicola* holds significance as an essential bamboo parasite renowned primarily for its production of hypocrellins from its fruiting bodies. Initially presumed to be a singular species within a monotypic genus, the systematic classification of this fungus has undergone several revisions over more than a century of investigation. Molecular genetic analyses have unveiled *S. bambusicola*‘s placement within Shiraiaceae, a newly established family within Pleosporales (Liu et al. 2013). Recently, a second hypocrellin-producing genus, *Rubroshiraia*, was added to Shiraiaceae (Dai et al. 2019). Both traditional and molecular identification methods remain necessary for characterising new species that yield hypocrellins. Notably, endophytic fungi sourced from bamboo have emerged as novel reservoirs of hypocrellin production. These fungal endophytes encompass a *Shiraia*-like endophyte group within the *Shiraia* genus, as well as endophytes from other genera like *Phaeosphaeria* and *Penicillium* (Meng et al. 2011; Li et al. 2012). Although hypocrellins produced by endophytes have predominantly been extracted from mycelia and quantified using chromatographic and spectroscopic techniques, future research endeavours are anticipated to focus on elucidating hypocrellin metabolites through nuclear magnetic resonance techniques to validate their production. Furthermore, beyond quantification in mycelium culture, efforts are warranted to elucidate and validate hypocrellin biosynthetic cluster genes, particularly in hypocrellins-producing endophytes not affiliated with *Shiraia*. Additionally, it is imperative to assess the latent pathogenicity and true endophytic nature of *Shiraia*-like endophytes. Thus, a systematic exploration of hypocrellins-yielding endophytes holds promise for the development of new fungal resources for effective PDT agents.

Currently, *Shiraia* mycelium cultures have emerged as promising alternatives for hypocrellin production. While the culture technology for solid plate and submerged liquid culture of *Shiraia* fungi is well established, hypocrellin yields in mycelium culture remain relatively low. Leveraging the sensitivity of fungal perylenequinone biosynthesis to biotic and abiotic stresses (You et al. 2008), various elicitors such as live bacteria, bacterial volatiles, fungal or bamboo polysaccharides, light or ultrasound exposure, surfactant treatment, and heat stress are artificially applied to mycelium cultures to simulate potential biotic or abiotic challenges, resulting in successful enhancement of hypocrellin production. Despite the recognised effectiveness of elicitation in promoting hypocrellin accumulation in mycelium cultures, the mechanisms underlying elicitor recognition and the interaction between elicitors and biosynthetic genes for hypocrellin production require further elucidation. Elicitation strategies can be integrated with other biotechnological approaches such as nutritional feeding, optimisation of culture conditions, medium renewal, and integrated processes (e.g. two-stage culture or two-phase processes) to achieve more significant enhancements in hypocrellin yields. Generally, combined elicitation is often more effective due to synergistic or potentiating effects compared to the use of single elicitors alone. Pretreatment with different elicitors at various stages or combined elicitation with signal molecules such as Ca^2+^, ROS, and NO is recommended to enhance hypocrellin production further. Recently, several genetically engineered *Shiraia* strains have been obtained by agrobacterium- or PEG-CaCl_2_-mediated transformation (Li et al. 2019a; Lu et al. 2024) and the CRISPR system for high-yielding hypocrellins (Deng et al. 2017; Bao et al. 2023). The overexpression of the carbon metabolism-related genes or central hypocrellin pathway genes could stimulate the biosynthesis of hypocrellins via increasing pathway flux (Gao et al. 2018a). However, there are no reports on eliciting these genetically engineered strains. To increase the simultaneous expression of key hypocrellin pathway genes, we suggest using a combination of appropriate elicitors. Moreover, elicitation may lead to the discovery of novel “hypocrellin-like” compounds or new photoactive perylenequinones with improved bioactivities, holding significant potential for PDT applications. While most previous studies on hypocrellin production in mycelium cultures have been conducted in shake-flasks with 25–50 mL medium, transitioning hypocrellin-yielding cultures from small shake-flasks to larger bioreactors is deemed essential for the biotechnological production of hypocrellins. Detailed investigations into crucial parameters for bioreactor operation, the stability of hypocrellin yield across batches, and overall production costs are warranted. It is envisioned that elicited mycelium culture of *Shiraia* endophytes will emerge as a commercially viable alternative for enhanced production of hypocrellins in bioreactors in the near future.
